# The women’s liberation movement, activism and therapy at the grassroots, 1968–1985

**DOI:** 10.1080/09612025.2018.1450611

**Published:** 2018-03-16

**Authors:** Sarah Crook

**Affiliations:** New College, University of Oxford, Oxford, UK

## Abstract

The women’s liberation movement was the impetus for the founding of new institutions of psychological and mental health care for women in the late 1970s and 1980s. This article draws upon the archive of one such site, based in Islington, North London, to explore the ways that members of the movement interacted with local politics and were attentive to racial and economic oppression. It demonstrates that consciousness-raising groups and feminist magazines made women’s distress visible and that this visibility led to the development of feminist critiques of mainstream psychiatric care. The critiques of mainstream provision laid the ground for grassroots interventions into women’s mental healthcare in the community.

The first edition of *Spare Rib*, published in 1972, invited women to comment on ‘what is a liberated woman’. Among the more skeptical responses (‘any ordinary sensible person who does not require “women’s lib” or any other do-gooders to tell her how to react’) were those that illustrated the contested meanings of liberation. One suggestion was that a liberated woman was ‘one who worked her way through and out of the psychological, social, emotional and intellectual limitations stamped on her by false role definitions and indifferent education.’^1^ The movement’s maxim of the ‘personal is political’ brought the social and emotional into view, while the emotional effects of oppression were summoned as evidence of the need for radical social change; their transformation was both a process during, and a product of, the advance towards liberation.^2^ ‘If the external situation subdues us, it is our consciousness that contains us’, the socialist feminist Sheila Rowbotham wrote in 1971.^3^ The feminist project therefore necessitated an examination of women’s consciousness, the excavation of the structures that regulated and labelled women’s emotions, and the creation of new sites of mental health care. It is the relationship between these—the critique of current structures and the formation of feminist healthcare at the grassroots level—that this article focuses on.

While in the 1990s historians were able to comment that ‘most histories of the second wave of the women’s liberation movement do not mention madness or “mental health” as areas of theoretical, political or practical intervention’, this has since developed into an area of greater interest, although this has largely expressed itself through an attentiveness to the theoretical and the political implications of the women’s liberation movement’s critique of mental health ideologies.^4^ Mathew Thomson has described how the British women’s movement positioned itself as an antagonist toward and an alternative to conventional psychiatry, for it ‘provided the women’s movement with a powerful enemy to identify themselves against’.^5^ As he demonstrates, though, the women’s movement was also able to appropriate the concept of the emotional and psychological self, with magazines like *Spare Rib* extending the ‘consciousness of consciousness to a broader audience’.^6^ The women’s movement was skeptical about the potential for women’s distress to be cured by traditional institutions concerned with the mind for these institutions were embedded in the very systems that perpetuated inequality. It moved beyond this critique, however, and in failing to foreground and historicize the institutions and sites of mental health care established by feminists we risk marginalizing the material and grassroots aims of the women’s movement.

This is particularly important in a movement that was characterized by and fully conscious of the importance of the local: the feminist magazine *Shrew* rotated responsibilities for editorship around the local groups of the London Women’s Liberation Workshop, reflecting the different interests, priorities and experiences of different local groups. Sarah Browne has recently observed that ‘more must be done in order to look at women’s liberation from the local and the small-group perspective’, for, as she explains, ‘the movement did not operate in isolation, but was shaped by the local communities from which it emerged.’^7^ Margaretta Jolly, too, has emphasized regional differences in the movement, while Sue Bruley has looked at feminist activism in five urban communities across England to draw attention to the diverse ways that groups responded to local concerns.^8^ Bruley rightly points out that the historiography of the movement is currently dominated by studies of London and calls for more attention to women’s centres, which, as she shows, have been overlooked by historians thus far.^9^ While this research is into a mental health project established in London, it demonstrates that London feminisms were far from a homogeneous mass—rather, they too need to be situated within and seen to be in dialogue with the particular concerns of the local population.

In response to the current historiographical emphasis on situating the local, I draw upon archival documents to explore the early history of the Maya Centre—then the Islington Women and Mental Health Project—to demonstrate that such establishments are integral to understanding feminist interventions into the health of their communities. The archive of the Islington Women and Mental Health Project was made available to me by the founders of the centre. The materials produced by the Project, I argue, show that feminists mobilized what would now be called an ‘intersectional’ understanding of the oppressions experienced by women in their community, placing the social and economic experiences of women at the heart of their analysis of mental health provision. Second, it highlights feminist resistance to the changes that were being made to mental healthcare at a national and local level in the 1980s. Beyond this, it also speaks to broader issues of women’s political activism at a local level. It was in this period that the barriers impeding women’s access to local politics in London were beginning to erode, and that issues affecting women’s life were—in part because of the Women’s Liberation Movement’s interventions—coming to play a greater role in local governments’ decision making.^10^ Although by the end of the 1970s and the beginning of the 1980s the women’s liberation movement had largely disbanded in its formalized, national sense, the local structures, relationships, intellectual affinities and political activisms that it had inspired had ongoing influence and importance. This, then, challenges our chronology of the movement and adds further evidence for the ineffective intellectual framework provided by the concept of ‘waves’ of feminism. This article demonstrates that the early approaches to women’s discontent in the women’s liberation’s most dynamic years acted as the foundation of the methods adopted and adapted by the Islington Women’s Mental Health Project. This article, then, first attends to the ways that the movement excavated and exposed women’s distress, transforming it into a political object.

The women’s movement demonstrated that discontent was political and could be a stimulus for activism around social change. Discontent, it argued, had been depoliticized by male-dominated mental health institutions, which had claimed expertise over the female experience. Kathie Sarachild, an American feminist credited as the pioneer of consciousness-raising, wrote in 1970 that ‘when we had hysterical fits, when we took things “too” personally, [we were] responding with our feelings correctly to a given situation of injustice’.^11^ The reclamation of women’s experiences and emotional states dissolved the boundary between the private and the political and provoked the establishment of new locations for the consideration of women’s health. In the women’s movement ‘pride of place’ was given to ‘the lived experience of mental illness, its connections with female construction of self, and to specific feminist therapies such as women’s consciousness raising groups’, argued L.J. Jordanova in 1981.^12^ ‘Bad feelings’ have been claimed by Melanie Waters to act as a ‘magnet’ around which women’s liberation’s political discourse developed in *Spare Rib*, the feminist magazine; here, there was a ‘virulent discourse of feeling.^13^ ‘Affect’, she argues, ‘is the current that animates and electrifies the complex web of personal, social and political identifications that spark between women in, through and beyond the pages of the magazine.’^14^ The webs of the personal, social and political were integral to the movement: as Lynne Segal asserted, ‘feminists always emphasised the importance of *the personal* and *the subjective,* the need for a total politics.’^15^ This ‘total politics’ drew connections between women’s emotions and their experiences of social and cultural structures. Sara Ahmed has argued that emotions do political work, contending that while pain does not underpin feminism, it can move women towards activism. ‘Pain’, she writes, ‘is moved into a public domain, and in moving, is transformed’.^16^ In this way, ‘If pain does move subjects into feminism, then it does so precisely by *reading* the relation between affect and structure, or between emotion and politics’.^17^ This brings to the fore questions about how and where the relationship between emotions and politics were exposed and transformed by the feminist movement of the 1970s and 1980s.

In the early years of the Women’s Liberation Movement a plethora of sites were established to extract feminist political meaning from personal feeling, including, among others, consciousness-raising groups, encounter groups and self-help groups. Women testified to the revelatory effects of this sort of engagement and this laid the groundwork for the creation of local organizations. In 1971 a woman going by M.F in the feminist magazine *Shrew* (1969–1974) wrote about her experience of an encounter group run by the women’s liberation movement.^18^ She found that ‘Feelings peeled back like the many skins of an onion until raw emotion was contacted’, and, ‘to my astonishment’ this emotion ‘turned out more often than not to be violent anger, or deeply held resentment which had been forcibly repressed.’^19^ The focus on revealing the psychologically repressed and the emotionally intimate was critical to the feminist political project.

While my focus here is on a single local site of psychological care, the landscape for this was initially mapped by the movement’s debates over psychiatry, consciousness-raising, and the possibility of feminist therapy. Put another way, before we can ask how women’s discontent was used to establish new sites of political activism and feminist therapeutic care, we must first ask: how did the women’s liberation movement make women’s discontent visible?

## Laying the ground work: critiquing the status quo

At the first Women and Mental Health Conference in London, held 22–23rd October 1977, a new definition of ‘mental health’ was arrived at: ‘mental health as self-determination, being able to choose to fit in or not fit in, to change or not.’^20^ This statement asserted autonomy and subjectivity in both the understandings of mental health and its treatment, but it also emphasized the extent to which mental health rested on socially-defined ideas of what it was to ‘fit in’, or to seek to change. Sandwiched between a notice about an action committee seeking the re-instatement of Maureen Colquhoun as Labour Party candidate for Northampton North and an article about abortion politics within the Labour Party, Ruth Wallsgrove noted that the ‘main issue’ that had emerged from the conference, aside from this definition, was the vexed question of if therapy could ever be feminist. ‘The argument’, she said, ‘is basically between those who believe that when we can’t cope we should take time to go through our problems with people who know what they are doing—that therapy makes us better able to go out and be political—and those that believe that personal one-to-one therapy is counter-revolutionary.’

The accusation that psychology was, as one American feminist framed it, a ‘link in the chain of women’s oppression’, drew upon evidence that women were disproportionally the targets of psychopharmaceutical intervention.^21^ This was made possible by the escalation of antidepressant production in the post-war years. The first of these post-war antidepressants, Miltown (meprobamate), named after the American town in which it was developed, was introduced to the mass market in 1955.^22^ Considered the first ‘designer drug’ within psychiatry, by the late 1950s it was widely prescribed and had entered the cultural lexicon.^23^ Librium (chlordiazepoxide) (1960) and Valium (diazepam) (1963) quickly followed, aiming to alleviate anxiety and depression.^24^ In England and Wales, in the half decade between 1965 and 1970, prescriptions for benzodiazepine tranquilizers rose by 110 per cent.^25^ In 1970 12.5 million prescriptions were issued in England and Wales for Librium, Valium and Mogadon.^26^ Women were found to be the recipients of these prescriptions: one 1971 study found that women were prescribed psychotropic drugs at double the rate of men.^27^

Therapists, too, were criticized for reinforcing rather than challenging the gender norms of the post-war period. Writing in *Shrew*, one female psychiatrist took aim at her profession. She noted that female patients presented with complaints that were usually about ‘keeping a man, changing a man or finding a man’; concerns that had been ‘reinforced’ by therapists.^28^ The Women’s Liberation Movement sought to challenge this. As Thomson has observed, feminist groups and publications were fecund for new ideas about how to disrupt and change conventional mental health care.^29^ The ideas were effectively circulated: the Psychology Group of the Women’s Liberation Workshop published an edition of *Shrew* dedicated to psychology in April 1972, explaining that it was initially established to ‘examine the liberating possibilities of psychotherapeutic techniques’ and to demonstrate the ways that ‘therapy is often used to trap women more tightly in their roles’, while setting out their aim to explain and explore the various models of psychotherapy that might be useful to the movement.^30^ They also encouraged those interested in establishing alternative forms of mental care provision to come to a self-help meeting in North London, where two groups emerged: the Women’s Re-evaluation Counselling Group, and the Women’s Self-Help Therapy Group.^31^ The desire to identify and locate the feelings and effects of oppression underpinned these modes of activism.

The women’s movement argued that the burdens generated by post-war gender ideologies were responsible for provoking women into discontent and distress. While domesticity was posited as the feminine ideal in the post-war period, the number of women in paid employment increased, driven in part due to the expansion of part time work.^32^ By 1974 women comprised nearly 41 per cent of the workforce, a shift that the TUC regarded as ‘one of the most important social developments of the past 30 years.’^33^ Mothers, too, were going out to work: according to the 1971 census, 588,600 women with children under five undertook paid work.^34^ Research showed that when women went out to work this duplicated rather than alleviated their domestic labour. Women were doing a ‘tandem of jobs’, Michael Young and Peter Willmott concluded in their influential study, *The Symmetrical Family*.^35^ This work was apt to be the ‘least psychologically rewarding’, and, at least normatively, comparatively financially expendable.^36^ Claire Langhamer has shown that the mother–child relationship and the marital bond assumed a new significance in the post-war years, ‘a fetishisation of emotional security stemming directly from the experience of war.’ ^37^ The effects and experience of the ideology of domesticity on educated women—those who had benefitted from the expansion of higher education in post-war Britain—was a topic of academic interest. Studies by Hannah Gavron, Viola Klein and Judith Hubback suggested that there was a tension between the conditions of domesticity, aspirations and women’s education.^38^ Indeed, Michelene Wandor wrote in a 1972 edition of *Spare Rib* that domesticity was the cause of women’s mental ill heath. Amidst an endless cycle of washing and childrearing, ‘No-one can understand that you’ve simply been driven mad and that it isn’t a condition that pills or therapy can cure’, she argued.^39^ The term ‘mad’ suggested a ‘state of dislocation in which you don’t know where or who you are’, and women became a ‘bewildered prize in the perpetual emotional tug of war between husband and children without time or space to worry about where you yourself fit in.’^40^ In this fraught situation, Wandor concluded, ‘No woman can emerge undamaged.’^41^ Patriarchy, it was argued, ‘ignores women’s emotional needs and punishes them for not conforming to their social role as caretakers of others.’^42^ Mainstream mental heath care, it was argued, was inadequate to deal with women’s distress, as this provision was grounded in patriarchal ideas about women’s selfhood.

Criticisms of the tenets of British psychological and mental health ideologies were informed by American critiques of the ‘psy’ disciplines..^43^ Naomi Weisstein’s deconstruction of psychological explanations of female nature was amongst the most important of these.^44^ First published in America, her essay ‘Kinder, Kuche, Kirche as Scientific Law: Psychology Constructs the Female’ was reprinted in London by Agitprop Literature in 1969. Weisstein claimed that ‘when we are about to consider the liberation of women, we naturally look to psychology to tell us what ‘true’ liberation would mean: what would give women the freedom to fulfill their own intrinsic natures’.^45^ She reflected upon the consensus between business and psychology (‘there’s a lot of money in “inner space”’) but was primarily concerned with debunking what she considered to be the false and limiting tenets of current psychological theories, suggesting that ‘the accidents of individual development or genitalia … has strangled and deflected psychology so that it is relatively useless in describing, explaining, or predicting humans and their behavior’, rendering psychology ‘less than worthless in contributing to a vision which could truly liberate.’^46^ She urged psychology to take an interest in the ‘social context’ of ‘inner traits’.^47^ This criticism was reiterated in the British women’s movement, where psychologists’ neutrality was roundly contested. An article in *Shrew* argued that psychologists ‘choose what subject they will investigate and how they will investigate it, and the criteria they will use for evaluation. And then society puts the study to its own use, so that typical middle-class families are taken as ideally adjusted to society … and women are kept in their place in home sweet home’*.*^48^ It went on to argue that social context was marginalized in favour of the more subjective ‘personality traits’: ‘Psychologists do their experiments on people who have been conditioned by society and then they tell us what we are like; and many women believe that’s how we must be, instead of trying to change’.

Others argued that mainsteam provision defined mental health in gendered ways. Phyllis Chesler’s *Women and Madness* was particularly emphatic on this point, demonstrating that ‘the ethic of mental health is masculine in our culture.’^49^ First published in America in 1972, extracts from Chesler’s book were published in *Spare Rib* in June 1973 prior to the text’s British release.^50^ Upon publication, *Spare Rib’s* review of the book concluded that ‘Beyond our bodies and our conditioning, we have minds. We can break patterns. Once our situation … is understood, we can go on to—what? At least, sanity.’^51^ Change, then, was an outcome of this feminist understanding; sanity was a product of feminist consciousness. How, then, was a feminist consciousness around mental health created?

## Making distress visible

Consciousness-raising diverged from, challenged and coalesced with conventional therapeutic approaches to women’s mental health. I argue here that it played a role in nurturing a feminist language that brought the self to the fore as well as acknowledging the oppressive effects of the social and political contexts in which women lived their lives. In this way, it laid the ground for the founding of grassroots women’s mental health projects in three ways: it brought local women together; it legitimized and recognized their experiences and distress; it prompted an acknowledgment that existing structures were not adequate. As Sue Bruley has noted, consciousness-raising developed at the very inception of the women’s movement as a mechanism for grassroots activism and recruitment.^52^ In November 1968 Kathie Sarachild of the New York-based Redstockings group read a paper (‘Consciousness-Raising: a Radical Weapon’) in Chicago that outlined the strategy for consciousness-raising that was later published and circulated in Britain.^53^ Conceived of as a conduit between large-scale social reform and individuals, women organized themselves into small groups, and held ‘a form of structured discussion in which women connected their personal experiences to larger structures of gender’.^54^ This sought to ascribe political meaning to the situation of the individual and to lay the ground for radical change. The practice sought to explore the relationship between personal experiences and structural oppression. ‘Consciousness-raising groups are a means whereby women share their often very personal experiences of discrimination and oppression, as a first step towards political understanding of it and resistance to it’, one feminist pamphlet published in 1976 explained.^55^ The ‘very personal’ stories shared were collectively validated and this nurtured political understanding.

This route to activism was not clear-cut, however, as the role of consciousness-raising as a therapeutic activity was emphasized as a critical part of the women’s movement’s broader strategy to give voice to women’s experience. Groups were founded on the premise that ‘sharing private suffering’ could be healing and emphasized personal experience as a path to political struggle.^56^ ‘In consciousness-raising’, the Psychology Group of the Women’s Liberation Workshop observed, ‘women discover the common nature of their problems and this understanding is channelled into a political perspective; the groups have a semi-therapeutic function in that they deal with the emotions aroused by the process of changing’.^57^ The political purpose of consciousness-raising distinguished it from therapy, although both were thought to have potential to make social structures visible through an interrogation of the individual psyche.^58^ Consciousness-raising quickly expanded beyond the small group, and editors of feminist magazines such as *Spare Rib* saw themselves as performing a consciousness-raising activity.^59^ In 1974 one reader wrote that ‘I read your magazine and I find I am right—life is hard, bloody hard. But the knowledge I am not alone and not crazy, makes me feel so much better and stronger’.^60^ This feeling of shared experience was reiterated in groups: one member of the Tufnell Park group recalled in *Spare Rib,* that the group served to assure her that ‘I need no longer consider myself a candidate for the ‘funny farm’, since so many of the women arrayed in that small sitting room, despite their surface differences, seemed to share what for so long I had believed to be my own idiosyncratic suffering.’^61^

As Bruley notes, however, local groups remained the preeminent way in which women were brought together to share their personal experiences within the movement.^62^ Local consciousness-raising groups were founded on the premise that articulating private emotional experiences could be transformed into the foundations of social critique. In her influential tract defining the parameters of ‘the personal is political’, Carol Hanisch claimed that it was ‘a political action to tell it like it is, to say what I really believe about my life instead of what I’ve always been told to say.’^63^ Political activism was closely bound to consciousness. In the wake of the famous demonstration against Miss World in 1969, the participants declared that ‘there is now a need to discipline our individual experiences of oppression and use them for a serious collective analysis. We must learn to use this pool of experience, this knowledge of oppression we all share, to reach in the future a new rigorous level of thought and action.’^64^

While the Women's Liberation Movement was vocal on the psychological consequences of women’s subordinate social position, the cures for the ensuing distress were fiercely disputed. More controversial than consciousness-raising groups, given their political and radical origins within the movement, was feminist therapy. In April 1978 this was given voice to in Issue 69 of *Spare Rib*. Sheila Jeffreys argued in her article ‘Against Therapy’ that there could be no such thing as feminist therapy, because the relationship duplicated the dynamic of patriarchy: ‘precisely the sort of authoritarian and hierarchical set-up which, as women, we are trying to get away from’.^65^ The very tools and techniques of therapy were oppressive, argued Jeffreys, who asserted that therapy had been developed by the male ruling class, and as such was not value free. She urged women to return to consciousness-raising, which was ‘the basis of the revolutionary struggle of women’, and provided a space for the ‘development of revolutionary anger and strength with others with whom we can take political action … its purpose is not to make an individual woman feel that she can cope better with her lot, but to make her feel that she need not cope, but must struggle.’^66^ The therapist was considered a conservative force that prevented women from rebelling against their oppression. Furthermore, the therapist perpetuated a culture of selfhood that fragmented women’s experiences. Jeffreys wrote that therapy was the ‘separation of the realm of mental health from the rest of our social and political lives’.^67^ Feminist therapy, it was suggested, focused on women’s internal lives rather than encouraging women to utilize their anger as a revolutionary political force.

Other women strongly disagreed. Writing in to *Spare Rib,* one correspondent argued that it was the individualistic nature of therapy that provided emancipation; ‘women have a right to choose something they want and maybe they are choosing therapy because it’s the *first* thing that’s come along which gives them something for themselves—and *why not?* I get sick to the stomach of hearing how I shouldn’t be so ‘introspective’—why the fuck *not, for once*?’^68^ This suggested that feminist therapy, in placing women’s experience at the heart of its techniques, subverted cultural ideologies of feminine self-effacement. Other members of the women’s liberation movement argued that feminist therapy could be complementary to feminist political activism. Stef Pixner, writing ‘For Therapy’ in *Spare Rib*, suggested that her experience in therapy had encouraged her to become ‘less depressed, more angry, more able to know what I need and act on it’.^69^ She noted that whilst many of the issues that brought women to therapy required political action, on a personal level women sometimes needed individual help to be able to find the strength to do this. In another article in *Spare Rib,* Frances Seton ‘found that the therapeutic process has given me a greater appreciation of political issues and motivation’.^70^ Although she initially viewed entering therapy as an admission of weakness, Seton found that ‘Having begun to trust the process, to understand the workings of the psyche and how to use the tools of therapy, I can … apply it to the larger social sphere.’ Through therapy she was able to ‘inform my political views with a new understanding of the political importance of psychology.’ Here, then, the therapeutic process was positioned as being one that facilitated personal transformation for political ends.

Tamsin Wilton, who helped to establish the telephone helpline at Bristol Crisis Service for Women during the late 1980s, claimed that through involvement in a feminist group she was given a ‘new and coherent conceptual framework’ for her experiences, and provided with a ‘*political* language for talking about madness’.^71^ Whereas in the early days of the movement women denounced psychological theories as constructing false dichotomies between male and female personalities—suggesting that all difference was culturally rather than biologically reproduced—by the later years of the movement there was a greater willingness to revisit and redeem some psychological theory.^72^ Two psychotherapists from the Women’s Therapy Centre in London, founded in April 1976, explained the shift as arising from the new types of knowledge developed in feminist spaces.^73^ In feminist organizations women ‘began to acknowledge that this distress might have a logic and life of its own even though its roots lay in the violence and oppression women experience within society.’^74^ Feminist therapy revealed the extent to which ‘nothing short of a restructuring of social arrangements is required if the conditions that give rise to women’s present psychological position are to change fundamentally.’^75^ This article now turns to an example of a women’s counselling centre in London that was established by members of the women’s liberation movement. This draws attention to how the emphasis on psychological wellbeing necessitated the creation of alternative provision, and demonstrates the practical as well as theoretical work that was done by the women’s movement.

## The Islington Women and Mental Health Project

The Islington Women and Mental Health Project (IWMHP) was established by a small collective of women who had been active in the women’s liberation movement and who had found the mental health provision in their local area to be deficient.^76^ The women’s liberation movement emerged in part from women’s social role, the founding members argued, as the movement ‘built upon the skills women have as a result of being the main carers in this society—our ability to relate and be attuned to others, to look after others and to feel such emotions as sadness and vulnerability and to ask for help.’^77^ The founders had come to know one another during two conferences organized by the London branch of the National Women and Mental Health Campaign, events which brought together patients and professionals within the mental healthcare system.^78^ From this, a national network emerged that sought to change mental health legislation and specialist groups were established to found crisis centres, support centres and refuges for women in emotional distress in local areas.^79^ The national campaign overlapped with other forms of feminist activism: the campaign could be contacted through Sisterwrite, the co-operative women’s bookshop that had opened in November 1978 and was based at 190 Upper Street in Islington.^80^ As Lucy Delap has demonstrated, feminist bookshops played an important role in the women’s movement. For women’s mental health campaigners, they provided both a site at which they could be contacted and also through which texts about women’s health could be distributed: the founders of the IWMHP credited Luise Eichenbaum and Susie Orbach, founders of the Women’s Therapy Centre, also based in Islington, as well as Phyllis Chesler (author of *Women and Madness,* 1972), Dorothy Dinnerstein (author of *The Rocking of the Cradle and the Ruling of the World*, 1978), and Nancy Chodorow (author of *The Reproduction of Mothering: Psychoanalysis and the Sociology of Gender,* 1978) with shaping their understanding of women’s psychology.^81^

The relationships between feminist organizations were overlapping. The founders of the Islington Women and Mental Health Project were central to the National Women and Mental Health Campaign: Mary Lynne Ellis, an art therapist and one of the Islington centre’s founders, was the author of the National campaign’s guiding statement. This statement argued that ‘mental health legislation and treatment are powerful weapons of social control which affect all people but women are a priority target.’^82^ Although this was demonstrated in women’s general prevalence as mental health patients, it rendered women of colour, gay women, working-class women and disabled women particularly vulnerable, she argued.^83^ ‘Behaviour that challenges patriarchy’ the statement pointed out, was ‘diagnosed as “crazy”’, and the treatment for this ‘aims at social readjustment and conformity to white, middle-class male standards of mental health’.^84^ Within this frame, the statement proposed that ‘“Breaking down” may be viewed as resistance rather than submission to male domination, and at the very least an attempt to escape from an intolerable life-situation’, and the purported cures ‘mask the real cause of a woman’s distress’.^85^ This distress, the statement argued, should instead be ‘viewed within its social context.’ Unsurprisingly, the Islington project, emerging from these London meetings of the National Women and Mental Health Campaign and with the active involvement of activists within it, shared this commitment to understanding women’s mental health concerns as arising from their social experiences.^86^ Not only this, but women had accumulated the skills and experience necessary to instigate social change: in a statement that outlined the project’s aims and values it was asserted that women were ‘the main organisers of the self-help movements, even those which are not self-consciously feminist’, positions that demonstrated women’s commitment to ‘supporting each other & taking action to improve the quality of our lives.’^87^

The Islington group initially established a telephone help line at Caxton House, 129 St John’s Way, N19, staffed by a volunteer. This volunteer had personal experience as a mental health patient, which underlined the conviction iterated through consciousness-raising that experience could be a form of feminist epistemology.^88^ This blurred the boundaries between service user and service provider within a feminist healthcare setting, diffusing the hierarchies enforced in mainstream healthcare. This blurring was not unusual and was occurring nationwide: the Bristol Women and Mental Health Network was established by three gay women who had met in a Bristol mental health hospital in 1986.^89^ From here the project expanded, and in 1983 the newly formed management committee successfully sought funding from Islington Council and was able to employ one worker as Project Coordinator—a post to which Brid Greally was appointed—and establish an office that remained in Caxton House. The building was shared with other organizations and itself has a rich history: the site that Caxton House Community Centre occupied had initially been developed as a workhouse in the early 1900s before it was taken over by London County Council in 1930. The original buildings were demolished in 1970 by the Greater London Council, with Caxton House Community Centre standing in their stead by 1976, the same year that it became a charity.^90^ Islington, a densely populated borough in inner London, remained a largely working-class borough in the post-war years, though from the mid-1950s house prices began to rise as the area drifted unevenly towards gentrification. The population was diverse as immigrants from the Commonwealth had settled in the area during the 1950s and 1960s.^91^ As a 1983 study of the area noted, the population was heterogeneous, with Indian, Turkish, Greek, Afro-Caribbean, Chinese, Italian and Vietnamese communities settled in the borough.^92^ The area also experienced high unemployment—in 1983 it was at 23 per cent. Women were revealed in this local survey to occupy a variety of positions: of the 65 women surveyed, 23 called themselves housewives, 14 were employed full time, five were employed part time, seven were unemployed, three were students, twelve were retired and one gave no information.^93^ This reflects that while Islington may appear to be a hub of feminist activity in this era—particularly in the founding of feminist therapy centres, given the Women’s Therapy Centre’s proximity—the area was in fact a mixing pot of cultures and classes.

The centre was alive to this convergence, bringing to the fore the ways that women’s identities were intersected by their economic and racialised experiences. While the project maintained a broad definition of mental health, which it suggested ‘about the quality of our lives … how much love/respect we get … it is about our ability to relate to others & to take care of ourselves’ this was structured within their systemic oppression: ‘women are considered inferior in this society & as we are at the receiving end of poverty, isolated in bad housing with small children & subject to sexism & racism, there are enormous stresses on our health.’^94^ The relationship between politics and health was articulated as fundamental to women’s experience. This was inflected by an understanding of how power acted on women, for, as the women running the centre pointed out, feminist work had ‘drawn connections between our oppression as women & being lesbian, being Black & being disabled & how these relate to our health.’^95^ Indeed, such experiences were made inevitable by political laxity: a society in which people ‘live in poverty, in poor housing conditions, subject to sexism and racism, ensures emotional and physical ill health.’^96^ Therefore the founders noted the importance of ensuring health care ‘is relevant to different ethnic groups, & to challenge racism in the health service.’^97^ As Natalie Thomlinson has argued, white feminists’ awareness of and activism around racism took the form of a variety of anti-racist practices which were sometimes clumsy, and that failed to challenge white women’s dominance over the movement as a whole.^98^ In the Islington project we can see further evidence that members of the women’s movement were not inattentive to the significance of race and that the theories developed during the 1970s continued to shape feminist activism into the 1980s, albeit manifested in local projects rather than articulated at national conferences.

The importance of housing to health and wellbeing was also being brought to the attention of the local council by residents themselves; one 1980 study of the Barnsbury Health Clinic conducted by Islington Community Health Council found that two thirds of the people interviewed said that housing affected their wellbeing—74 per cent of whom claimed that it was adversely affecting their health.^99^ The health service as it stood was considered to be complicit in women’s distress, as it failed to take heed of the rubric in which women lived their lives and instead treated the symptoms of oppression as illness. By contrast to this medicalization, the centre proposed that psychological distress could be seen as ‘essentially our protest and potentially empowering’.^100^ Ellis and Greally wrote that ‘we totally refute the concept of “mental illness”, a biological notion that carves a split between our distress and the rest of our lives’.^101^ In its current state, psychiatry did ‘not have the ability to give us better mental health’.^102^ They were explicit about the campaign’s origins in the emotional domain made visible by feminist consciousness: ‘Our anger at the present mental health service continues to fuel our campaign for better services.’^103^ One of the aims of the centre was to provide for women for whom other mental health services were difficult to access.^104^ By 1987 the IWMHP had secured funding from the Islington Institute for a tutor to train women to run the telephone helpline and had established a crèche for the clients, a series of ‘Coping with Stress’ courses, a women and depression group, an art therapy group, a drop-in service, and offered short-term psychodynamic counselling.^105^ This is illustrative of the dynamic provision developed by those with a feminist consciousness of mental health issues.

A study of the project also refocuses our attention on the ways that feminist mental health services interacted with the shifts in mental health services on a local level. As a study conducted by Islington MIND and Islington Community Health Council into local mental health provision 1983 found, the area was one in which organizations were run with ‘innovation and energy’, but many schemes were ‘unsupported and fragmented’.^106^ The report noted that the early 1980s were a period of transition for mental health services; the large hospitals established by the Victorians were argued to have allowed contemporary patients to become ‘isolated from their original communities and entrenched in the total institution of the hospital’.^107^ The report warmly claimed that this had become ‘clearly recognized and services are looking outwards back to the community.’^108^ Measures had been put in place that would ‘enable large numbers of patients to be cared for in the community’ and which ‘have also prevented people from being admitted to the hospitals’, in line with the national government policy which was increasingly oriented towards community-based care.^109^ At this time Islington’s population of 167,000 was catered for by the Whittington Hospital, which had a psychiatric unit of 90 beds and 50 day places, and Friern Hospital, which offered rehabilitation, psychogeriatric provision and day hospital facilities: 300 patients from Islington attended, and the further 900 were drawn from neighbouring Camden and Haringey.^110^ As the report noted, though, the majority of mentally ill people relied on community-based care.^111^ It was this push towards community-based care that drew the ire of the IWMHP, expressed in a 1985 riposte to Islington Health Authority’s consultation on district-based mental health services.

The document highlighted the gendered assumptions and effects of Community Care.^112^ First, it argued that the consultation obscured the extent to which women’s mental health issues were provoked by social and cultural structures. This, it argued, ‘serves to deny the social origins of distress, instead locating the “illness” solely with in the individual’, moving them to ask ‘why a document on “community care” continues to divorce personal experience from the wider social context where poverty, bad housing conditions, racism and sexism are the major sources of distress.’^113^ Second, the project pointed out that women’s unpaid labour was used to compensate for the deficiencies of other services. Indeed, the authors charged that Community Care ‘exploits us in its demand that women take on this caring, further endangering our mental health and offering no forms of support that are really responsive to women’s needs’.^114^ A survey conducted by Islington Community Health Council between October and December 1977 into the needs of families and relatives caring for an elderly person at home confirmed this accusation.^115^ The report characterized the 35 houses included in the survey as:

Mostly small—usually one daughter, herself in late middle age, caring for an elderly mother. There are several cases of people living with daughters and sons-in-law (but not sons and daughters-in-law) and a few families caring for elderly couples. In nearly all the families only one person, the daughter, does the caring. Only five households contain young children and three of these consist of a lone mother caring for a small child and an elderly parent.^116^

This gendered labour remained unremarked upon in the report, however, and none of the eleven recommendations formulated by the health council related to the disproportionate responsibilities borne by women. The women in the Islington project were not alone in recognizing the implications for women of the reorganization of services: in 1991 an article by Helen Smith in *Feminism & Psychology* argued that it was ‘crucial that the political debate is revealed for what it often is—a hypocritical concern for high quality care which masks the hidden agenda of establishing power and saving money.’^117^ Community care, feminists at both the national and the grassroots reflected, was predicated on the assumption that women would step into the gap left by the withdrawal of the state: as the women working at the Islington project demonstrated, the relationship between gender roles, feminism and the politics of mental healthcare needed unpicking at the local level. This local group demonstrates the ways that women’s activism in mental health was locally grounded as well as nationally articulated.

## Conclusion

After the acrimonious final national women’s liberation movement conference of 1978 and into the 1980s, members increasingly attested that the emotional climate had shifted. As ‘Janella’ argued in the lesbian magazine *Artemis* in 1983, ‘It used to be fashionable to be always ranting … about the state of the world. Now we look at it with an attitude of amused tolerance. It isn’t *our* civilisation that’s coming apart at the seams. It’s patriarchy.’^118^ The idea of liberation had been a ‘great trap’, Janella decided, for in ‘the sixties and seventies there was this cult of political commitment, anger and hope. We dreamed of utopias and ended with dust and ashes. We were fools to expect anything better of patriarchy.’^119^ Anger, however, had been mined and mobilized to entrench this political commitment, forging a relationship between anger and hope: anger at how things currently were; hope that it could be made better. This hope that things could be made better focused feminist activity on both the material and the psychological. The experiential knowledge that was developed in consciousness-raising groups and expressed in feminist publications provoked the creation of new sites of psychological care at the local level. By looking at how this excavation was transformed into grassroots activism we can see how the movement engaged with practical political issues through the lens of women’s mental health; this, in turn, expands our understanding of how some members of the women’s liberation movement understood marginality, race and economic oppression in their local communities. The conceptual apparatus and tools of the early women’s liberation movement allowed feminist critiques to be taken forward and for feminist activism to be enacted on the local level, providing sites of interaction with local politics and national policy agendas. It also exposes the ways that a feminist lens on mental health demanded that material and structural oppressions be challenged, too; the psychological was political.

## Notes

1 In Our Own Write (1972) *Spare Rib*, 1, p. 5.

2 Carol Hanisch (1970) ‘The Personal is Political’, in Shulamith Firestone & Anne Koedt (Eds) *Notes from the Second Year: women’s liberation* (New York: Radical Feminism), pp. 76–78.

3 Sheila Rowbotham (1971) ‘Women’s Liberation and the New Politics’, *Mayday Manifesto*, 2(6), p. 2.

4 Tamsin Wilton (1995) ‘Madness and Feminism: Bristol crisis service for women’, in Gabriele Griffin (Ed.) *Feminist Activism in the 1990s* (London: Taylor and Francis), pp. 28–40, p. 32. See, for example, Nancy Tomes (1994) ‘Feminist Histories of Psychiatry’, in Mark Micale & Roy Porter (Eds) *Discovering the History of Psychiatry* (Oxford: Oxford University Press), pp. 348–383.

5 Mathew Thomson (2006) *Psychological Subjects: identity, culture, and health in twentieth–century Britain* (Oxford: Oxford University Press), p. 281.

6 Ibid. p. 279.

7 Sarah Browne (2014) *The Women’s Liberation Movement in Scotland* (Manchester: Manchester University Press), p. 63.

8 Margaretta Jolly (2012) ‘Recognising Place, Space and Nation in Researching Women’s Movements: sisterhood and after’, *Women’s Studies International Forum*, 35(3), pp. 144–146.

9 Sue Bruley (2016) ‘Women’s Liberation at the Grass Roots: a view from some English towns, c.1968–1990’, *Women’s History Review*, 25(5), pp. 723–740.

10 Jim Barry (1991) *The Women’s Movement and Local Politics: the influence on councillors in London* (Aldershot: Avebury).

11 Kathie Sarachild (1970) ‘A Program for Feminist “Consciousness-Raising”’, in *Women’s Liberation: notes from the second year, major writings of radical feminists*, pp. 78–80, p. 78.

12 L. J. Jordanova (1981) ‘Mental Illness, Mental Health: changing norms and expectations’, in Cambridge Women’s Studies Group (Ed.) *Women in Society: interdisciplinary essays* (London: Virago), pp. 95–114. p. 97.

13 Melanie Waters (2016) ‘Yours in Struggle: bad feelings and revolutionary politics in Spare Rib’, *Women: A Cultural Review*, 27(4), pp. 446–465, p. 447.

14 Ibid. p. 448. For more on feminist magazines, see Laurel Forster (2016) ‘Spreading the Word: feminist print cultures and the Women’s Liberation Movement’, *Women’s History Review*, 25(5), pp. 812–831.

15 Lynne Segal (1979) ‘A Local Experience’, in Sheila Rowbotham, Lynne Segal & Hilary Wainwright (Eds) *Beyond the Fragments: feminism and the making of socialism* (London: Merlin Press), pp. 157–210, p. 164. Emphasis in original.

16 Sara Ahmed (2004) *The Cultural Politics of Emotion* (Edinburgh: University of Edinburgh Press), p. 173.

17 Ibid. p. 174.

18 M.F. (1971) Encounter, *Shrew*, 3(6), pp. 14–16, p. 14.

19 Ibid. p. 14.

20 Ruth Wallsgrove (1977) ‘Choosing to Fit In … Or Not Fit In’, *Spare Rib*, 65, p. 13.

21 Susan Williams (1975) ‘Women’s Psychology: mental illness as a social disease’, Presented to women and mental health symposium, 19 May 1975, Seattle, Washington (Seattle: Radical Women Publications), p. 1.

22 Jonathan Gabe (1991) ‘Introduction’, in Jonathan Gabe (Ed.) *Understanding Tranquilliser Use: the role of the social sciences* (London: Routledge), pp. 1–12, pp. 3–4.

23 Christopher M. Callahan & German Berrios (2005) *Reinventing Depression: a history of the treatment of depression in primary care, 1940–2004* (Oxford: Oxford University Press), pp. 106–109.

24 Gabe, ‘Introduction.’

25 Ibid.

26 Ibid.

27 Peter Parish (1971) ‘The prescribing of psychotropic drugs in general practice’, *Journal of the Royal College of General Practitioners* 21(92), pp. 1 –77.

28 A Psychiatrist’s View (1972), *Shrew*, 4(2), p. 3.

29 Thomson, *Psychological Subjects*, p. 280.

30 Editorial (1972), *Shrew*, 4(2), pp. 1–2.

31 Carol Morrell (1973) ‘On Not Being the Hostess with the Mostest’, *Spare Rib*, 16, pp. 37–38.

32 See Dolly Smith Wilson (2006) ‘A New Look at the Affluent Worker: the good working mother in post-war Britain’, *Twentieth Century British History*, 17(2), pp. 206–229.

33 Report of a TUC Working Party (1976) *The Under–Fives* (London) p. 11.

34 Ibid. p. 13.

35 Michael Young & Peter Willmott (1975) *The Symmetrical Family* (Harmondsworth: Penguin), p. 101.

36 Ibid.

37 Claire Langhamer (2017) ‘Feelings, Women and Work in the Long 1950s’, *Women’s History Review*, 26(1), pp. 77–92, p. 78.

38 See Helen McCarthy (2016) ‘Social Science and Married Women’s Employment in Post–War Britain’, *Past & Present*, 233(1), pp. 269–305, and Helen McCarthy (2016) ‘Women, Marriage and Paid Work in Post–War Britain’, *Women’s History Review*, 26(1), pp. 46–61.

39 Michelene Wandor (1972) ‘Family Everafter’, *Spare Rib* 5, pp. 10–13, p. 12.

40 Ibid.

41 Ibid.

42 Bristol Women’s Therapy Centre (1981) ‘Talking with Women in Bristol’, *Spare Rib*, 102, pp. 6–8, p. 8.

43 Lynne Segal (1987) *Is the Future Female? Troubled thoughts on contemporary feminism* (London: Virago), p. 119.

44 Segal, *Is the Future*, p. 119.

45 Naomi Weisstein (1969) *Kinder, Kuche, Kirche as Scientific Law: psychology constructs the female* (London: Agitprop Literature), p. 1.

46 Ibid. p. 1.

47 Ibid. p. 2.

48 The Dangers of Professionalism (1972) *Shrew*, 4(2), pp. 4–5, p. 4.

49 Phyllis Chesler (1973) *Women and Madness* (New York: Avon Books), p. 69.

50 Phyllis Chesler (1973) ‘Women and Madness: the modern paradox’, *Spare Rib*, 12, pp. 25–30.

51 Carol Morrell (1974) ‘Women and Madness’, *Spare Rib*, 26, pp. 39–40, p. 40.

52 Sue Bruley (2013) ‘Consciousness Raising in Clapham: women’s liberation as “lived experience” in south London in the 1970s', *Women’s History Review*, 22(5), pp. 717–738.

53 Kathie Sarachild (c.1968) ‘Consciousness–Raising: a radical weapon’, in *Feminist Revolution* (New York: Random House), pp. 144–150.

54 Baxandall & Gordon, *Dear Sisters*, p. 13.

55 Bodleian library, M91B638 Women’s Liberation, an Introduction (1976), p. 7.

56 Jordanova, Mental Illness, p. 97.

57 Editorial (1972), *Shrew*, 4(2), pp. 1–2, p. 1.

58 Luise Eichenbaum & Susie Orbach (1985) *Understanding Women* (Middlesex: Penguin), p. 26.

59 Marsha Rowe (1982) ‘Introduction’, in Marsha Rowe (Ed.) *Spare Rib Reader: 100 issues of women’s liberation* (Middlesex: Penguin), pp. 13–22, p. 19.

60 Ann Scott (1974) ‘Why is Your Magazine so Depressing?’, *Spare Rib* 34, p. 8.

61 Belsize Lane Women’s Group (1982) ‘Nine Years Together’, in Marsha Rowe (Ed.) *Spare Rib Reader: 100 issues of women’s liberation* (Middlesex: Penguin), pp. 561–575, p. 566.

62 Bruley, Consciousness-Raising in Clapham.

63 Carol Hanisch (1970) ‘The Personal is Political’, in *Notes from the Second Year: women’s liberation major writings of the radical feminists* (New York), pp. 76–78.

64 Women’s Liberation Workshop (1969), *Shrew*, p. 2.

65 Sheila Jeffreys (1987) ‘Against Therapy’, in Sue O’Sullivan (Ed.) *Women’s Health: a spare rib reader* (London: Pandora Press), pp. 142–145, p. 143.

66 Ibid. pp. 143–144.

67 Ibid.

68 Claire Betti (1978) ‘Letters Forum’, *Spare Rib*, 71, p. 36.

69 Stef Pixner (1978) ‘For Therapy’, *Spare Rib*, 69, pp. 20–21.

70 Frances Seton (1976) ‘Opening Myself to Change’, *Spare Rib*, 44, pp. 30–32, p. 32.

71 Tamsin Wilton (1995) ‘Madness and Feminism: Bristol crisis service for women’, in Gabriele Griffin (Ed.) *Feminist Activism in the 1990s* (London: Taylor and Francis), pp. 28–40, p. 28.

72 Lynne Segal (1987) *Is the Future Female? Troubled thoughts on contemporary feminism* (London: Virago), pp. 117–122. See Juliet Mitchell (1974) *Psychoanalysis and Feminism* (Harmondsworth: Penguin).

73 Ernst & Maguire, Introduction, *Living with the Sphinx*, p. 8.

74 Ibid.

75 Luise Eichenbaum & Susie Orbach (1987) ‘Separation and Intimacy: crucial practice issues in working with women in therapy’, in Sheila Ernst & Marie Maguire (Eds) *Living with the Sphinx: papers from the women’s therapy centre* (London: Women’s Press), pp. 49–67, p. 49–50.

76 The author wishes to extend her deep thanks to Mary Lynne Ellis and Brid Greally for sharing these documents with her. Islington Women and Mental Health Project, (undated; c. 1985) *Community Care: betrayal of women*; Mary Lynne Ellis (2014) *Maya Centre: early years – Islington Women and Mental Health Project*; Brid Greally (1985) *What is Islington Women & Mental Health?*; Mary Lynne Ellis & Brid Greally (undated, c. 1985), *What we Women Want in Terms of a Mental Health Service*.

77 Mary Lynne Ellis & Brid Greally (undated, c. 1985), *What we Women Want in Terms of a Mental Health Service*, p. 1.

78 Mary Lynne Ellis, *Statement for National Campaign,* p. 1.

79 Ibid.

80 Women & Mental Health, ‘Women & Mental Health Meeting’, (undated); (1978) ‘Announcement: Sisterwrite’, *Women’s Studies International Quarterly*, 1(4), p. 403. For more on feminist bookshops, see Lucy Delap (2016) ‘Feminist Bookshops, Reading Cultures and the Women’s Liberation Movement in Great Britain, c. 1974–2000’, *History Workshop Journal*, 81(1), pp. 171–196. Sisterwrite also provided space for the London Feminist History Group to meet. See Anna Davin (1980) ‘The London Feminist History Group’, *History Workshop Journal*, 9(1), pp. 192–194.

81 Mary Lynne Ellis (2014) *Maya Centre: early years – Islington Women and Mental Health Project*.

82 Ellis, *Statement for National Campaign*, p. 1.

83 Ibid.

84 Ibid.

85 Ibid.

86 Ellis & Greally, *What we Women Want*, p. 1–2.

87 Ibid.

88 Ellis, *Maya Centre: early years*, p. 1.

89 Wilton, ‘Bristol Crisis Service’, p. 35.

90 Claire Hardiman, Our Story—A History of Caxton House and the St. John's Way Site, http://www.caxtonhouse.org/history.html.

91 P. R. Williams (1976) ‘The Role of Institutions in the Inner London Housing Market: the case of Islington’, Transactions of the Institute of British Geographers, 1(1), pp. 72–82; Peter Congdon (1990) ‘The Analysis of Small Area Social Change’, *Progress in Planning*, 34, pp. 189–290.

92 Brenda Neale (1983) *An Investigation into the Learning Needs of Adults in Islington*, Joint Learners Advisory Service Research Project, p. 2–3.

93 Ibid. p. 20.

94 Brid Greally (1985) *What is Islington Women & Mental Health?*, p. 4.

95 Ibid.

96 Ellis & Greally, *What we Women Want.*

97 Greally, *What is.*

98 Natalie Thomlinson (2012) ‘The Colour of Feminism: white feminists and race in the Women’s Liberation movement’, *History*, 327(97), pp. 453–475.

99 Islington Community Health Council (1981) *Survey of Barnsbury Health Clinic*, p. 3.

100 Ellis & Greally, *What we Women Want*, p. 1.

101 Ibid.

102 Ibid.

103 Greally, *What is.*

104 Ellis, *Maya Centre*, p. 1.

105 Ellis, *Maya Centre*, p. 1; personal communication with Ellis.

106 Islington Community Mental Health Council and Islington MIND (1983) *Good Practices in Mental Health in Islington* (London), p. 6.

107 Ibid. p. 7.

108 Ibid.

109 Ibid.

110 Ibid.

111 Ibid.

112 Mary Lynne Ellis & Brid Greally (1985) *Community Care: Betrayal of women* (London).

113 Ellis & Greally, *Community Care*, p. 1.

114 Greally, *What is.*

115 The Elderly Person at Home in Islington: report of a survey carried out between October and December 1977 by Islington Community Health Council, London, 1978.

116 Ibid. p. 1.

117 Helen Smith (1991) ‘Caring for Everyone? The implications for women of the changes in community care services source’, *Feminism & Psychology*, 1(2), pp. 279–292, p. 281.

118 Mother and Daughter (1983) *Artemis* 2, pp. 14–15, p. 15. (emphasis in original)

119 Ibid. p. 14.

